# Biodiversity and distribution of zoobenthos in the ecological water replenishment area of the Yellow River estuary coastal wetland revealed by eDNA metabarcoding

**DOI:** 10.1371/journal.pone.0315346

**Published:** 2024-12-18

**Authors:** Gang Xie, Jie Lan, Jinhui Liang, Qidong Wang, Xiaoqiang Cao, Yanlong Wang, Chongyang Ren, Huaqing Liu, Jian Zhang

**Affiliations:** 1 State Environmental Protection Key Laboratory of Land and Sea Ecological Governance and Systematic Regulation, Jinan, China; 2 Shandong Academy for Environmental Planning, Jinan, China; 3 College of Safety and Environmental Engineering, Shandong University of Science and Technology, Qingdao, China; 4 Institute of Yellow River Delta Earth Surface Processes and Ecological Integrity, Shandong University of Science and Technology, Qingdao, China; 5 School of Geographical Environment, Shandong Normal University, Jinan, China; Central University of South Bihar, INDIA

## Abstract

The coastal wetland of the Yellow River Estuary, one of China’s largest wetlands, is essential for biodiversity conservation and ecosystem services. Ecological water replenishment, a typical wetland restoration measure in the Yellow River Delta, has significantly impacted the habitat of zoobenthos, which are critical indicators of ecosystem health and water quality. However, the community characteristics of zoobenthos in this coastal wetland are poorly understood. This study utilized eDNA metabarcoding to assess the diversity and community structure of zoobenthos in the ecological water replenishment area of Yellow River Estuary Coastal Wetland. Zoobenthos from 174 families were identified, with 307 species recognized at the generic level, significantly more than those identified through traditional morpho-taxonomic approaches. Salinity emerged as a crucial factor in shaping these ecosystems. Contrary to expectations, in this study, brackish water exhibited the lowest species richness compared to freshwater and seawater, which may be attributed to local environmental stressors and fluctuating salinity conditions in the Yellow River Estuary. Environmental factors such as salinity, organic matter, and nutrient elements significantly influence the composition and distribution of zoobenthos. Specifically, cations, particularly Mg^2^⁺ and Ca^2^⁺, have a more substantial impact on zoobenthos than anions. Our results provide crucial information on zoobenthic biodiversity within ecological water replenishment areas, offering insights into the ecological dynamics and environmental factors shaping zoobenthos communities under ecological management.

## 1. Introduction

The Yellow River Delta wetland, located on the Bohai Sea coast in northeastern Shandong Province, is the broadest, most complete, and youngest wetland ecosystem in the warm temperate zone (with an average annual temperature of 11.7–12.6°C). Since the Yellow River’s diversion to the Bohai Sea in 1855, the continuous interaction between the river and the ocean has driven geomorphological evolution, creating a diverse ecosystem that supports rich species diversity [1]. It acts as a key indicator of ecological health in the Yellow River Basin and functions as a vital germplasm repository and source of marine biodiversity in the Bohai Sea region [2]. According to the white paper "Biodiversity Conservation in the Yellow River Delta", the coastal wetland hosts 1,145 species of wild plants, 373 species of birds, and 512 species of insects. The wetland is crucial for biodiversity, and supports various fish, invertebrates, and plant species. In addition, it provides essential ecological services, such as water purification, flood regulation, carbon sequestration, and shoreline stabilization. Ongoing conservation efforts focus on pollution control, sustainable water resource management, and habitat protection to ensure the wetland’s ecological and economic sustainability. Recent studies on macrozoobenthos in coastal ecosystems have demonstrated the utility of eDNA metabarcoding for environmental monitoring. He et al. (2021) analyzed benthic impacts from salmon farming, revealing that eDNA metabarcoding effectively captures shifts in macrofaunal community structures, including changes in alpha diversity and species composition, under varying environmental stressors. Their research highlights that macrofaunal taxa like polychaetes exhibit distinct responses to organic enrichment, with eDNA data providing enhanced resolution in detecting such changes compared to traditional morphological methods [3]. Similarly, Cordier et al. (2017) demonstrated the application of supervised machine learning with eDNA metabarcoding data to predict the ecological status of marine environments. Their study showed that eDNA-derived benthic data not only aligns well with conventional biotic indices but also offers a scalable and cost-effective solution for routine benthic monitoring. This method overcomes challenges associated with unassigned sequences in taxonomic databases, making it a robust tool for future ecological assessments [4]. Despite these advancements, the understanding of aquatic biodiversity, particularly related to zoobenthos, in specific regions such as the Yellow River Delta remains limited. Expanding research efforts using eDNA metabarcoding will be crucial to bridge these knowledge gaps and ensure the sustainable management of these ecologically valuable systems.

Zoobenthos, aquatic organisms that reside on the bottom of water bodies for most or all of their lives, are critical ecological indicators in coastal wetlands [5,6]. Their presence, abundance, and diversity provide valuable insights into sediment and water quality. Zoobenthos play an essential role in nutrient cycling by decomposing organic matter and recycling nutrients like nitrogen and phosphorus, which support primary production and maintain ecological balance [7]. As a fundamental part of the aquatic food web, they are a primary food source for fish and bird species, directly impacting higher trophic levels [8]. Their activities, such as burrowing, affect sediment structure and stability, influencing the distribution of other organisms [9]. Additionally, some zoobenthos species can bioaccumulate contaminants, aiding in bioremediation and pollution management [10]. Different zoobenthic species have distinct ecological roles in coastal wetlands. Amphipods, such as *Gammarus* spp., can enhance nutrient availability through detritus consumption and sediment aeration [11]. Polychaetes were observed to increase sediment permeability and support nutrient cycling via bioturbation [12]. Bivalves, like *Mytilus edulis*, play roles in improving water quality and stabilizing sediments through filtration [13]. These examples indicate that understanding the population structure and distribution characteristics of zoobenthos is crucial for maintaining the health and functionality of coastal wetland ecosystems.

There are significant differences in the living habits of zoobenthos. For instance, the larvae of Chironomidae are often found in nutrient-poor sediments where they consume detritus, enhancing nutrient availability and supporting their population growth [14]. In contrast, the *Capitella capitata complex* thrive in organically enriched environments, often associated with pollution, benefiting from high organic matter availability for their feeding and reproductive activities [15]. Salinity also plays a crucial role in the distribution of zoobenthic species. For example, the Baltic clam, *Macoma balthica*, is adapted to low-salinity environments and is commonly found in brackish waters with varying salinity levels [16]. Conversely, some species of blue mussel are more tolerant of higher salinity levels and are typically found in marine environments [17]. With the replenishment of ecological water, salinity and nutrient elements in the coastal wetlands of the Yellow River Delta show gradient variations [18]. It is speculated that ecological water replenishment lead to spatial differences in the distribution of zoobenthos within the coastal wetland [19].

Traditional benthic surveys, relying on morpho-taxonomic approaches, are labor-intensive, time-consuming, and require significant expertise [20]. These methods face challenges such as low throughput, ambiguities in taxonomic identification, and significant difficulties in sample preservation, which can affect both the accuracy and reliability of the results. In contrast, the rapid advancement of high-throughput sequencing has positioned molecular taxonomy as a promising tool for biodiversity studies. These DNA-based techniques identify taxa using specific genome fragments, known as "DNA barcode" regions [21]. Metabarcoding employs high-throughput sequencing to detect multiple species from complex samples. Environmental DNA (eDNA) metabarcoding targets environmental samples, offering significant advantages such as high throughput, non-invasiveness, and the ability to detect species at low abundance levels with high specificity [22]. Several studies have shown that eDNA metabarcoding can outperform traditional methods in terms of detecting species richness and identifying cryptic or rare species that may not be easily detected using morphology-based approaches. For instance, a study in freshwater ecosystems revealed that eDNA detected 1.1 to 2 times more species than traditional sampling methods, especially in environments where physical sampling might be limited due to habitat complexity [23,24]. Similarly, in freshwater bivalve monitoring, eDNA was found to detect species that were missed in conventional surveys, highlighting its potential for early detection of invasive species [25]. Advances in sediment sampling techniques and DNA extraction methods are enhancing the efficiency and reliability of eDNA applications in zoobenthos surveys.

This study aimed to investigate the community structure of zoobenthos in the ecological water replenishment area of the coastal wetland in the Yellow River Estuary using eDNA metabarcoding. Key environmental factors influencing community structure were identified through correlation analysis. The findings of this study could enhance our understanding of the biodiversity and distribution patterns of zoobenthos in the ecological water replenishment area of Yellow River Estuary Coastal Wetland but also provide valuable insights for biodiversity conservation, habitat protection, and ecosystem management. By identifying key environmental drivers, the study contributes to more effective conservation strategies, such as targeted habitat restoration and the design of management practices that support the preservation of aquatic biodiversity in this unique ecosystem.

## 2. Materials and methods

### 2.1. Study area and sample collection

The Yellow River Estuary Coastal Wetland, located on the south coast of Bohai Bay and the west coast of Laizhou Bay in China (between 117°31′-119°18′E and 36°55′-38°16′N), is a significant ecological region with a semi-humid and semi-arid warm-temperate continental monsoon climate. This area experiences four distinct seasons, an average annual temperature of 12.1°C, and annual rainfall between 530 and 630 mm, predominantly in the summer months. Prevailing winds from the south-southeast and east influence its weather patterns.

**Fig 1:**
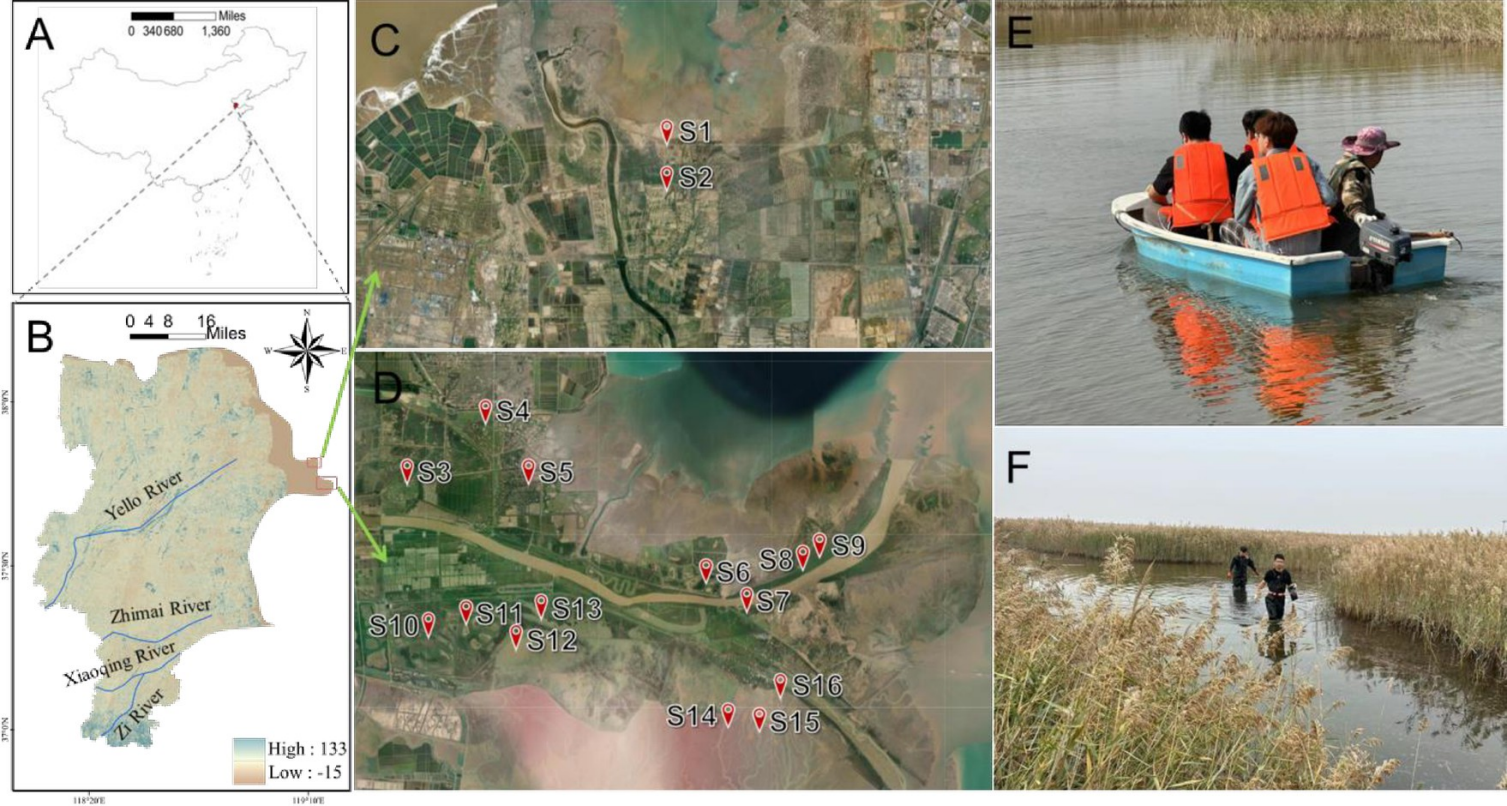
Geographic location (A) and elevation (B) of the Yellow River delta. locations of 2 sampling sites in the One Thousand and Two Management Station (C) and 14 sites in the Shandong Yellow River Delta National Nature Reserve (D); habitat conditions during sample collection (E) and (F). (Base map from USGS; url: https://www.usgs.gov/tools/national-map-viewer).

In October 2023, sediment and water samples were collected from 16 sites within the ecological water replenishment area (Fig 1). Two sites were managed by the One Thousand and Two Management Station of the Yellow River Delta, covering the old course of the Yellow River. The remaining 14 sites were within the Yellow River Delta National Nature Reserve. The longitude and latitude coordinates of the 16 sampling sites were shown in S1 Table. Three parallel samples were taken at each site: approximately 1.0 kg of surface sediment and 1.0 L of water from the same points [4]. The sediment weight was estimated by collecting a known volume of sediment, which was later confirmed by weighing in the laboratory. The sediment was collected using a bottom sampler (LB-TC1001, Lubo, Qingdao, China). Pebbles, twigs, shells, and other debris were carefully removed from the sediment, and the sample was thoroughly mixed before being placed in a sterile sampling bag. Water samples were collected using sterilized high-density polyethylene (HDPE) bottles. The bottles were rinsed with site water three times before collecting the final 1.0 L sample at the same locations. To prevent cross-contamination, sterile gloves were worn during both the sampling and analysis processes, and gloves were changed between each sample. All samples were kept on ice during transport and analyzed immediately upon arrival at the laboratory.

### 2.2. Ethical statement

This study utilized environmental DNA (eDNA) technology and did not involve the capture or experimentation of animal subjects. All field sampling was conducted in accordance with relevant legal and ethical guidelines and with approval from the Shandong Yellow River Delta National Nature Reserve Administration Committee.

### 2.3. Physicochemical parameters analysis

The life of zoobenthos is closely related to the surrounding water environment; therefore, the physicochemical indices of the collected water samples were analyzed with emphasis [26]. Part of the physicochemical indices were measured in situ. For example, dissolved oxygen (DO), oxidation-reduction potential (ORP), and temperature (T) were determined using a portable DO meter (HQ40d, Hach, Colorado, USA). Electrical conductivity (EC), total dissolved solids (TDS), salinity (SAL) and pH were measured using an EC meter (DDB-303A, Leici, Shanghai, China), a TDS meter (WQM01H-3, Bit Atom, Shenzhen, China), a SAL meter (WS-100, Bingmu, Shenzhen, China) and a pH meter (pH-100, Lichen, Shanghai, China), respectively. The total organic carbon (TOC) content was measured using a TOC analyzer (TOC-L, Daojin, Kyoto, Japan). The total phosphorus (TP) and ammonium ion (NH_4_^+^) contents were determined using a UV-Visible spectrophotometer (UV1800, Shimadzu, Japan) in accordance with standard procedures (APHA, 2005). The sulfate ion (SO_4_^2−^), sodium ion (Na^+^), magnesium ion (Mg^2+^), potassium ion (K^+^), and calcium ion (Ca^2+^) concentrations were quantified using an ion chromatography analyzer (HD-TLS51, HORDE, Shandong, China).

### 2.4. DNA extraction and sequencing

Studies have demonstrated that directly extract sediment eDNA can reliably characterize zoobenthic diversity and community composition [27,28]. Sediment eDNA was extracted from approximately 0.25 g of sediment for each sample using the E.Z.N.A.® Soil DNA Kit (Omega Bio-tek, Norcross, GA, USA) according to the manufacturer’s protocols [27]. To ensure robustness, triplicate subsamples were analyzed independently for each site. The V4 regions of the zoobenthos 18S ribosomal RNA gene were amplified by PCR (95°C for 2 min, followed by 25 cycles of 95°C for 30 s, 55°C for 30 s, and 72°C for 30 s, with a final extension at 72°C for 5 min) using primers F04mod F: 5’-GCTTGWCTCAAAGATTAAGCC-3’ and F04mod R: 5’-CCTGCTGCCTTCCTTDGA-3’. Each sample was tagged with a unique eight-base barcode sequence [27]. PCR reactions were performed in triplicate in 20 μL mixtures, each containing 4 μL of 5× FastPfu Buffer, 2 μL of 2.5 mM dNTPs, 0.8 μL of each primer (5 μM), 0.4 μL of FastPfu Polymerase, and 10 ng of template DNA. Amplicons were extracted from 2% agarose gels and purified using the AxyPrep DNA Gel Extraction Kit (Axygen Biosciences, Union City, CA, USA) following the manufacturer’s instructions [24]. To prevent sample DNA contamination, equipment such as forceps, scissors, pipettes, and measuring spoons were cleaned with 10% bleach and rinsed with ultrapure water before and between each use. To avoid laboratory contamination, separate rooms were used for pre- and post-PCR processes [29]. Purified PCR products were quantified using a Qubit® 3.0 (Life Technologies, Invitrogen). Twenty-four amplicons with unique barcodes were pooled in equal amounts. This pooled DNA was used to construct an Illumina paired-end library according to Illumina’s genomic DNA library preparation protocol. The amplicon library was sequenced (2 × 250 bp) on an Illumina MiSeq platform (Shanghai BIOZERON Co., Ltd) following standard protocols.

Raw FASTQ files were demultiplexed using in-house Perl scripts based on barcode sequences for each sample, following these criteria: (i) reads were truncated at any site with an average quality score below 20 over a 10 bp sliding window, and truncated reads shorter than 50 bp were discarded. (ii) exact barcode matching was required, allowing for a 2-nucleotide mismatch in primer matching; reads with ambiguous characters were removed. (iii) only sequences with overlaps longer than 10 bp were assembled; non-assembled reads were discarded. A reference database was generated using ECOPCR by extracting sequences from the EMBL database (version 143) with F04mod primers and the NCBI classification database. Initial taxonomic assignments were made using ECOTAG [30]. Each assigned zoobenthic sequence was manually verified through BLAST searches in the NCBI nucleotide sequence database and compared against local species surveys following previously described criteria [19]. Only sequences with ≥98% identity to GenBank entries were included in subsequent analyses to ensure accurate species-level assignments. Human sequences were excluded from all analyses.

### 2.5. Statistical and multivariate analyses

Zoobenthic invertebrate abundance was analyzed with detrended correspondence analysis (DCA) to determine gradient lengths, revealing values below 3, which justified the use of Redundancy Analysis (RDA) to explore relationships between community composition and environmental variables [31]. Community composition differences were visualized using Nonmetric Multidimensional Scaling (NMDS), with PERMANOVA assessing significant differences across sampling sites [32,33]. The Shannon–Wiener index and ANOVA were used to analyze alpha diversity indices and community composition differences [34]. The Kruskal–Wallis test was used only when data did not meet ANOVA assumptions [35]. A co-occurrence ecological network was constructed using OTUs with > 0.1% relative abundance, analyzed through RMT-based methods [36,37].

## 3. Results

### 3.1. Environmental parameters

The water quality analysis from various sampling sites (S1 to S16) reveals significant variability with ecological water replenishment, along with several noteworthy relationships among the physical and chemical parameters (Table 1). EC and TDS exhibit a strong positive correlation, as TDS measures the dissolved ions contributing to EC. For example, site S16 shows both the highest EC (70400 ± 5895 μS/cm) and TDS (38250 ± 1665 mg/L), while site S2 has the lowest values for both parameters (EC: 889 ± 1 μS/cm, TDS: 444 ± 2 mg/L). Additionally, SAL also positively correlates with EC and TDS. This suggests that salinity is a major contributor to the dissolved solids that influence conductivity. Sulfate concentrations also contribute to the overall dissolved solids and conductivity. For example, site S16, with high sulfate levels (1024 ± 5 mg/L), exhibits correspondingly high TDS and EC. The concentrations of cations such as Na^+^, K^+^, Mg^2+^, and Ca^2+^ are positively correlated with each other and with TDS, reflecting the common ionic composition of the coastal water samples. DO generally shows an inverse relationship with temperature, with higher temperatures often resulting in lower DO levels due to decreased oxygen solubility in warmer water. Nutrient levels, such as NH_4_^+^-N and TP, show variable correlations with organic and inorganic parameters. Higher NH_4_^+^-N levels, indicating potential pollution sources such as anthropogenic activities, are observed at sites S8 and S9, correlating with high TOC levels.

**Table 1:** Water quality parameters of samples taken from the Yellow River Estuary Coastal Wetland.

Sampling site	S1	S2	S3	S4	S5	S6	S7	S8	S9	S10	S11	S12	S13	S14	S15	S16
**T (°C)**	19.20±0.55	20.40±0.05	21.40±0.45	21.80±0.00	20.10±0.10	21.90±0.15	21.50±0.40	21.10±0.16	22.30±0.32	19.90±0.00	20.60±1.05	19.80±0.00	21.80±0.25	19.50±0.12	19.60±0.11	19.50±0.40
**pH**	7.85±0.67	8.11±0.10	8.31±0.20	8.46±0.23	8.32±0.00	9.56±0.17	8.68±0.17	8.58±0.17	8.32±0.26	8.61±0.02	8.62±0.06	8.32±0.04	8.94±0.18	8.45±0.00	8.29±0.20	8.31±0.35
**DO (mg/L)**	5.34±1.76	8.08±0.05	7.92±1.43	9.96±2.22	8.91±0.06	10.76±0.24	10.27±0.00	10.82±0.35	10.12±0.09	9.30±0.40	8.58±0.47	7.32±0.21	9.96±0.78	9.41±1.97	9.41±0.92	9.41±1.51
**ORP (mv)**	87.7±139.5	79.8±29.6	100.9±13.3	118.2±4.3	193.7±0.1	163.0±10.0	165.7±13.6	183.5±1.3	150.2±5.6	149.2±10.1	144.7±0.7	149.6±7.7	159.5±7.1	144.4±11.9	144.6±23.0	144.2±18.9
**EC (μS/cm)**	26545±9167	889±1	1303±122	2178±819	5921±403	1983±343	1636±42	39870±232	44670±4	1660±159	1224±42	1265±39	891±10	68470±113	52740±148	70400±5895
**TDS (mg/L)**	13270±4582	444±2	651±61	1067±421	2965±204	991±171	867±45	19930±123	22330±5	830±80	614±22	632±20	445±5	37280±279	29420±72	38250±1665
**SAL (mg/L)**	1050±272	44±0	62±5	103±40	296±3	113±16	77±3	1920±123	2180±13	79±5	72±1	74±0	55±3	2630±49	2690±7	3030±80
**NH**_**4**_^**+**^ **-N (mg/L)**	1.21±0.03	0.09±0.00	0.19±0.24	0.67±0.24	1.10±0.01	0.27±0.21	0.13±0.03	2.03±0.92	2.42±0.95	0.45±0.02	0.32±0.06	0.33±0.00	0.07±0.02	0.01±0.35	0.02±0.20	0.01±0.17
**TP (mg/L)**	0.038±0.010	0.004±0.044	0.043±0.012	0.048±0.020	0.053±0.002	0.004±0.025	0.038±0.005	0.098±0.012	0.078±0.100	0.038±0.000	0.033±0.000	0.048±0.002	0.033±0.007	0.112±0.001	0.107±0.006	0.068±0.017
**TOC (mg/L)**	23.26±4.67	4.36±1.73	6.37±0.79	5.90±2.73	8.65±0.35	8.50±1.35	6.39±0.23	11.92±0.95	11.67±0.42	8.28±0.26	7.45±0.44	6.88±0.26	5.39±0.09	4.69±1.47	4.45±2.32	4.61±3.38
**SO**_**4**_^**2-**^ **(mg/L)**	487.5±21.5	40.3±18.7	147.2±10.9	188.0±39.5	256.4±25.3	188.8±6.1	173.6±4.9	1024.7±15.2	1168.7±41.6	161.9±10.1	144.6±11.6	164.0±1.6	125.7±55.0	1952.8±64.8	1902.3±9.3	2267.8±31.2
**Na**^**+**^ **(mg/L)**	3056±731	183±51	171±26	262±122	842±45	382±61	26±88	4921±205	5580±260	223±18	118±33	238±6	131±19	6287±140	8616±33	8154±199
**K**^**+**^ **(mg/L)**	102.6±49.9	13.3±1.6	11.7±1.3	11.0±3.3	36.8±2.1	20.3±1.9	4.0±5.6	212.7±19.2	260.6±26.2	13.7±0.3	13.5±0.3	16.5±1.3	11.5±1.1	328.6±0.2	449.2±1.5	405.8±6.7
**Mg**^**2+**^ **(mg/L)**	419.2±105.0	39.8±10.2	42.9±5.0	48.8±29.0	119.0±5.8	2.6±26.0	8.7±19.6	546.0±26.3	627.8±29.2	49.0±1.8	46.2±1.2	52.0±2.6	32.6±5.1	748.5±28.1	996.5±6.2	605.5±28.9
**Ca**^**2+**^ **(mg/L)**	213.3±58.6	104.4±22.3	48.1±1.6	46.4±23.1	107.7±4.6	36.2±3.1	9.3±14.0	268.9±3.2	302.3±5.1	49.0±6.0	37.1±1.5	33.1±5.4	35.5±0.3	315.5±18.1	427.8±3.4	271.7±20.7

### 3.2. Zoobenthos community compositions and distributions

Zoobenthos comprising 20 phyla, 31 classes, 73 orders, and 174 families were identified through sediment eDNA metabarcoding (S2 Table). The primary phyla include Annelida, Arthropoda, Gastrotricha, Nematoda, Ostracoda, and Platyhelminthes. These phyla are divided into classes such as Chromadorea, Clitellata, norank, Ostracoda, and Rhabditophora. Each class further divides into orders, including Chaetonotida, Chromadorida, Monhysterida, and Plectida. These orders lead to specific families such as Aphanolaimidae, Chaetonotidae, Chromadoridae, Dalyelliidae, Limnocytheridae, Monhysteridae, Naididae, Schizocytheridae, Typhloplanidae, and Xyalidae, among others (S1 Fig).

**Fig 2:**
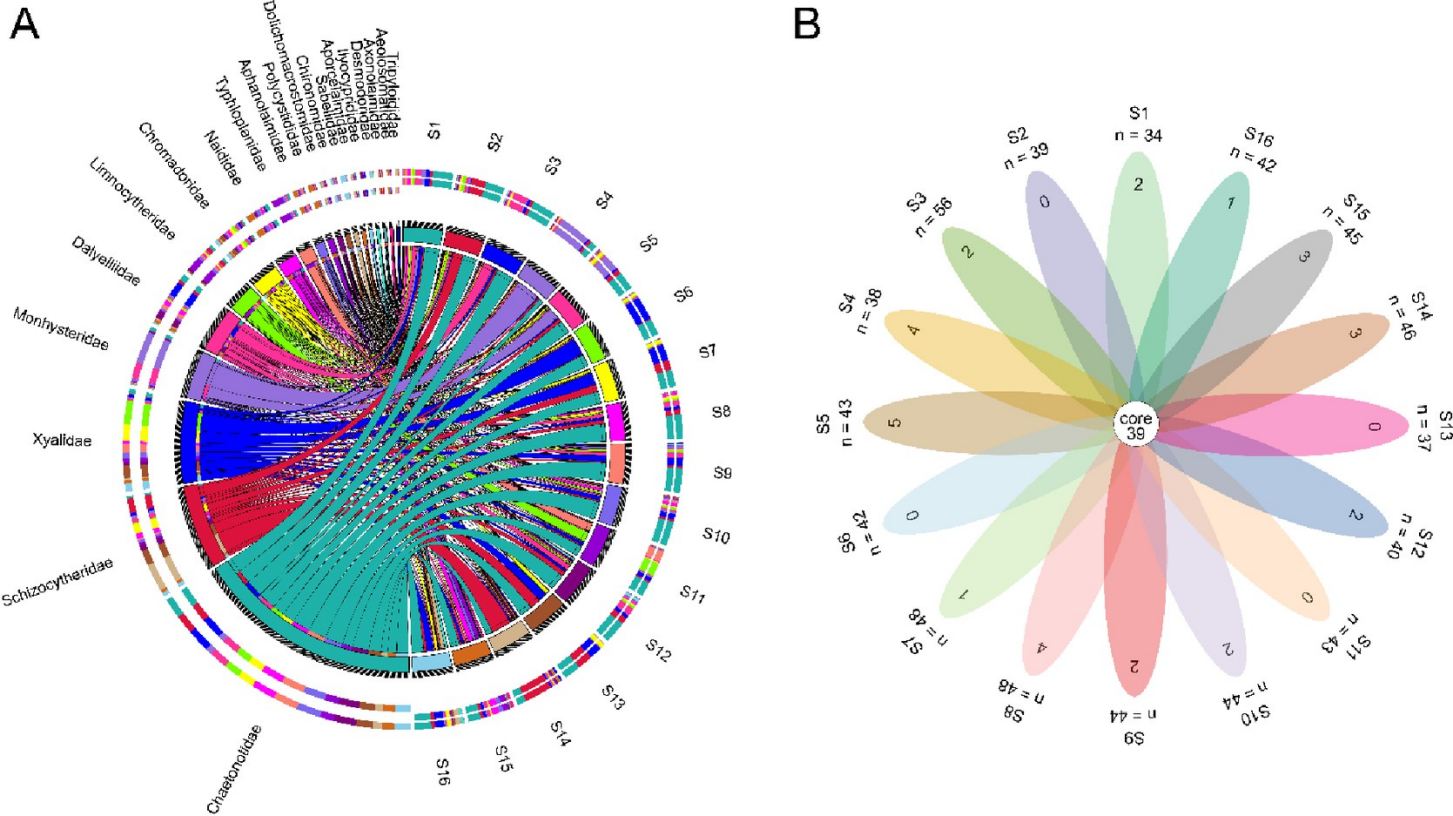
Zoobenthic community compositions (A) and population statistics (B) across different sampling sites in the Yellow River Estuary Coastal Wetland. The circos plot displays the 20 most abundant zoobenthic families. In the petal plot, values within the petals represent the number of zoobenthic families unique to each sample, and the sum of the value outside the petal and the core value indicates the total number of families.

The circos chart in Fig 2A highlights significant differences in zoobenthic species composition across sampling points S1 to S16. Each sampling point is represented by a segment in the outer circle, with color-coded bars denoting the relative abundance of various species families. The inner chords illustrate transitions and interactions between species groups across these points. Notable variations are observed, with Limnocytheridae and Typhloplanidae being more prevalent at point S11. Additionally, Xyalidae are prominent at points S6 and S7, while Schizocytheridae show higher presence at points S2, S13, and S14. These patterns suggest distinct ecological niches or environmental conditions influencing species distribution at different sampling points. The Venn diagram in Fig 2B illustrates the species composition differences at the family level across 16 sampling points. The central "core 39" represents 39 species common to all sampling points. Each petal indicates the number of unique species for each respective sample. For example, sample S3 has 56 unique species, S14 has 46, and both S9 and S8 have 48 unique species each. The total number of species in each sample is calculated by adding the core value (39) to the unique species count. Thus, S3 contains 95 species (39 core + 56 unique), and S14 has 85 species (39 core + 46 unique). Although the data on shared species between different points is not shown, the diagram effectively highlights both the diversity and commonality of species across the sampling points, emphasizing the ecological richness and variation within the zoobenthic ecosystem.

### 3.3. Relationships between environmental parameters and zoobenthos communities

**Fig 3:**
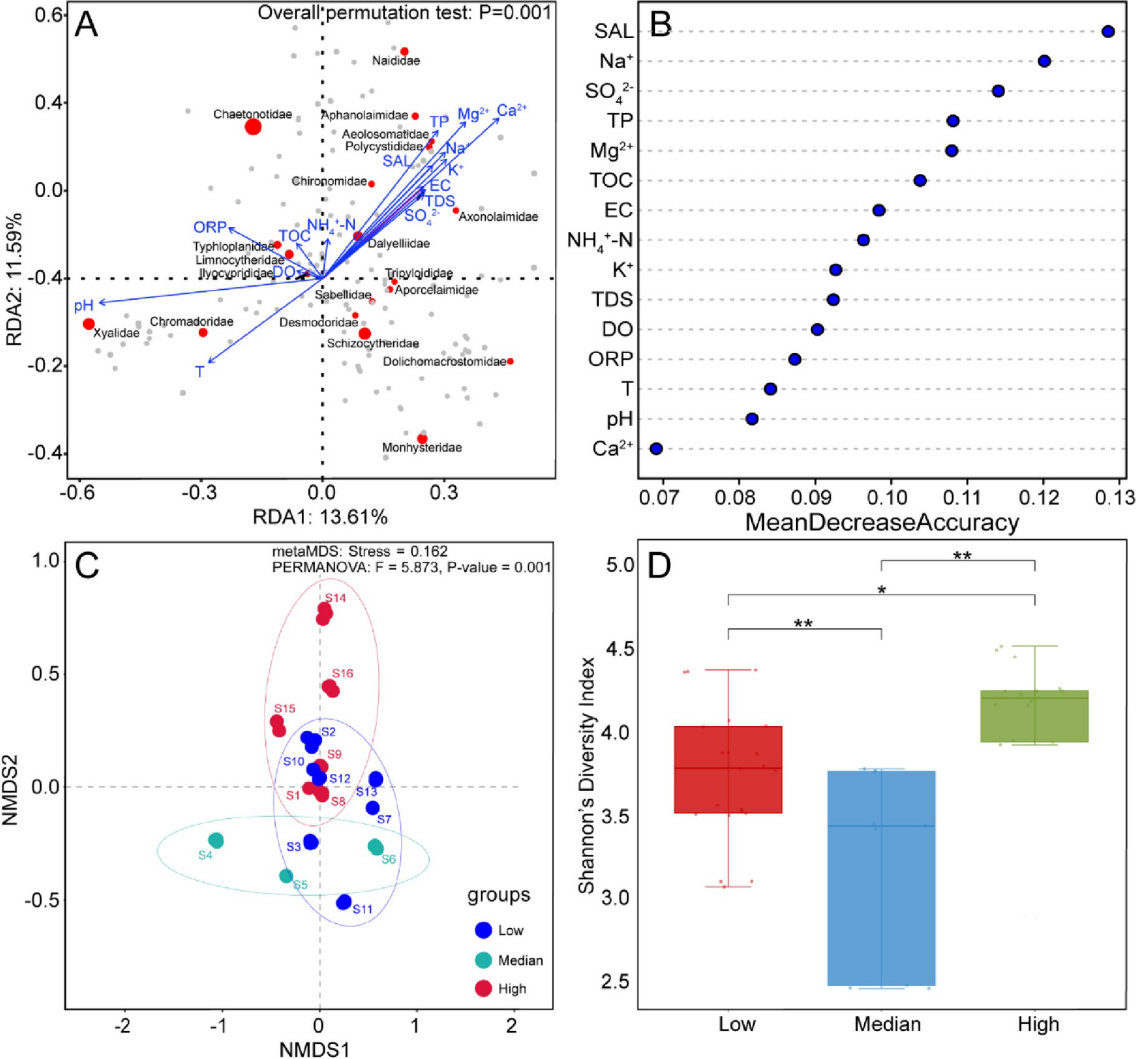
RDA analysis illustrating the relative abundance of zoobenthic families at each sampling site in relation to environmental factors (A). Scatter plots showing the prediction of environmental variable importance using random forests (B). β-diversity (C) and α-diversity (D) of zoobenthic communities among samples with varying salinity.

The RDA plot illustrates the relationship between zoobenthos and various environmental factors (Fig 3A). Blue arrows represent environmental factors, with their direction and length indicating the gradient and strength of influence on zoobenthos. The overall permutation test (P = 0.001) confirms that these environmental factors significantly impact the zoobenthos community. Salinity, represented as a comprehensive index of cationic and anionic concentrations, plays a crucial role in shaping these communities. Higher salinity levels are associated with families such as Naididae, Aphanolaimidae, Aeolosomatidae, and Polycystididae, suggesting these families prefer nutrient-rich, saline environments. Conversely, Limnocharidae and Hyocopridae prefer areas with low salinity and higher oxygen levels. Additionally, a random forest regression analysis was utilized to determine the relative importance of various environmental factors influencing zoobenthos (Fig 3B). Each point on the graph represents the importance score of a specific factor, with higher scores indicating a greater influence on the presence, abundance, or health of these organisms. The trend in the graph indicates that certain environmental factors, particularly salinity, significantly shape the distribution and abundance of different zoobenthic families in aquatic ecosystems.

The NMDS plot shows the β diversity of zoobenthic communities across different salinity conditions (Fig 3C). Statistical analysis confirms significant differences between high and low salinity groups (PERMANOVA: F = 5.873, P = 0.001). The metaMDS stress value of 0.162 indicates a good representation of the data. The low salinity group (blue) is distinct and primarily located in the lower left quadrant, while the high salinity group (red) is separate and situated in the upper region. The median salinity group (cyan) is positioned between the low and high groups, indicating a transitional community structure. Fig 3D illustrates the α diversity of zoobenthos, measured by Shannon’s diversity index, under three different salinity conditions. Statistical analysis reveals significant differences in diversity among all salinity groups. The low salinity condition exhibits a relatively high median diversity, indicating a rich variety of zoobenthic species. In contrast, the median salinity condition has the lowest median diversity, suggesting that intermediate salinity levels are associated with reduced species diversity. The high salinity condition shows the highest diversity, indicating that extreme salinity levels, particularly high salinity, support a greater variety of zoobenthic organisms.

**Fig 4:**
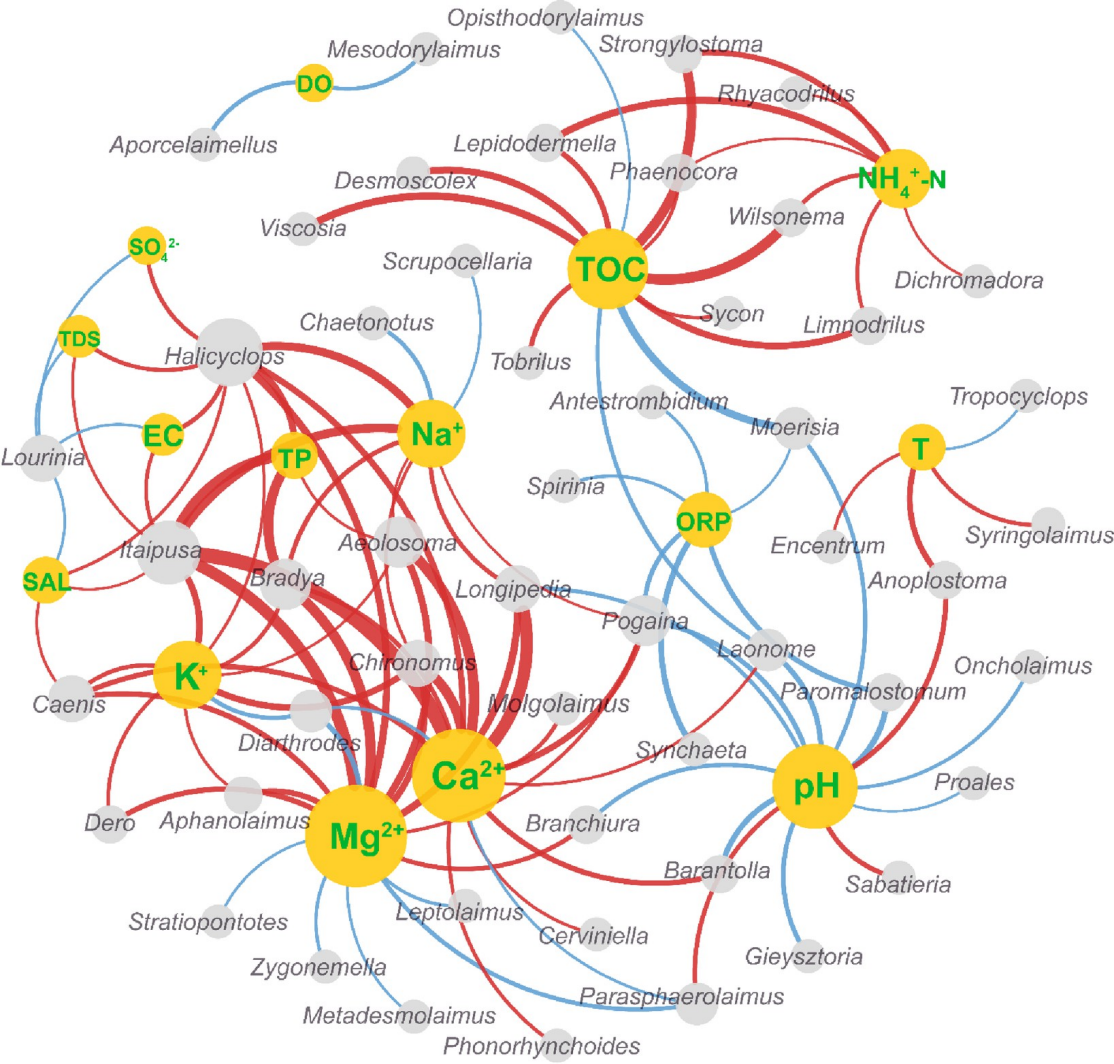
Co-occurrence network of environmental parameters and individual zoobenthic genera. Red lines represent positive correlations, while blue lines indicate negative correlations. A larger node area signifies a greater number of associated species or environmental factors, and a thicker line denotes a higher correlation coefficient.

The ecological network relationships between complex environmental factors and various zoobenthic genera is presented in Fig 4 The nodes represent environmental factors (yellow) or benthic animal genera (gray), and the edges (lines connecting the nodes) depict the interactions between them. Red lines denote positive correlations, whereas blue lines indicate negative correlations. The thickness of the lines reflects the strength of these relationships. Key environmental factors such as Ca^2^⁺, Na⁺, TOC, and pH exhibit numerous strong correlations with various zoobenthic genera, suggesting a significant influence on the composition and distribution of these communities. In contrast, factors like TDS and EC have fewer and weaker connections, indicating a lesser impact on zoobenthic genera.

## 4. Discussion

### 4.1. eDNA metabarcoding and zoobenthic diversity

In the field of zoobenthos identification using eDNA metabarcoding, several primers are employed to target specific gene regions for amplifying DNA fragments. The 18S rRNA gene primers, like F04mod F and F04mod R, are highly advantageous due to their conservation across eukaryotic species, offering broad applicability and reliable detection of diverse zoobenthos [38]. COI gene primers, such as LCO1490 and HCO2198, are widely used for their ability to amplify DNA from a broad range of invertebrates, making them suitable for many benthic organisms [39]. Lastly, the 12S rRNA gene primers, such as MiFish-U/E, while primarily used for fish, can also effectively target certain benthic species, adding to the versatility of eDNA studies in aquatic systems [40]. These primers vary in specificity and range, with 18S rRNA and COI being more universally applicable across zoobenthos, and 12S tailored for specific groups.

In the coastal wetland of the Yellow River Estuary, zoobenthos from 174 families were identified using 18S rRNA gene primers, with 307 species identified at the generic level. In contrast, traditional morpho-taxonomic approaches in previous studies identified only 60 zoobenthic families in the Yellow River Estuary Coastal Wetland, with further categorization at the genus or species level hindered by the need for specialized expertise in morphological classification [41]. Based on the bioinformation provided by eDNA metabarcoding, the zoobenthic genera were categorized according to their connectivity within and among modules. This analysis identified several key species as connectors: *Eldenia*, *Chironomus*, *Tholymis*, *Gomphonema*, *Caenis*, *Proales*, *Strombidium*, *Sinistrostrombidium*, *Mononchus*, *Paracerior*, *Tubulanus*, *Micrura*, and *Robbea* (S2 Fig). These genera exhibit high among-module connectivity, indicating their crucial role in linking different modules within the network. *Chironomus* is a genus of midges in the family Chironomidae, commonly known as non-biting midges. These insects inhabit a variety of freshwater environments and can thrive in both clean and polluted water bodies. The larvae are a significant food source for fish and other aquatic organisms. Due to their sensitivity to pollutants and ease of culture in the laboratory, *Chironomus* species are frequently used in environmental monitoring and toxicological studies. The presence of Chironomidae in the Yellow River Estuary Coastal Wetland has been confirmed using traditional morpho-taxonomic approaches, whereas the research does not provide identification at the generic level [19]. For example, *Eldenia*, a genus of flatworms in the family Provorticidae, primarily inhabits brackish water environments, particularly in marine benthic zones such as shores and intertidal areas [42]. This genus has been documented in regions such as Germany, specifically in the brackish waters of the Northern Hemisphere. The presence of *Eldenia* and *Chironomus* species, which thrive in brackish environments, highlights the adaptability of certain zoobenthos to fluctuating salinity conditions in estuarine systems.

**Fig 5:**
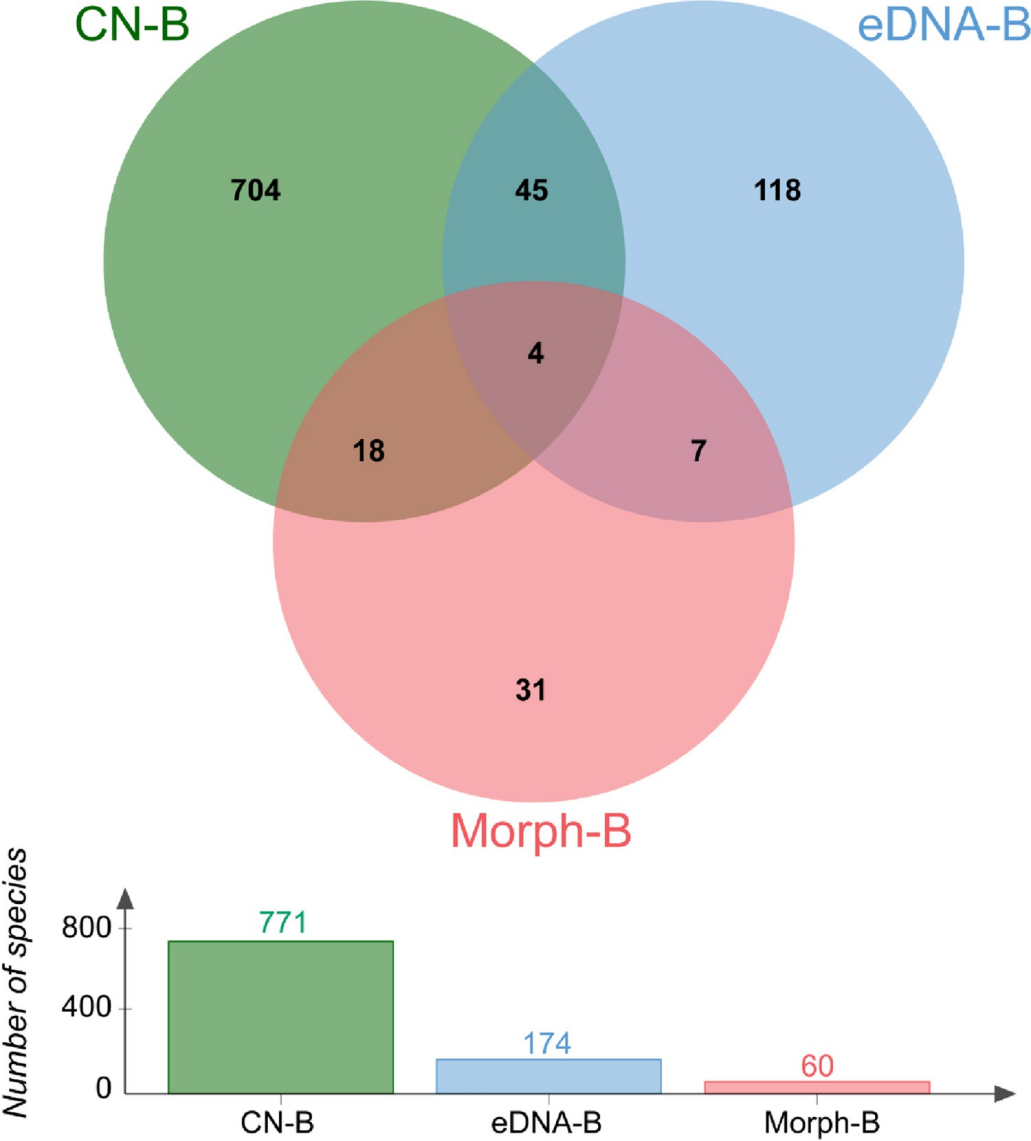
Comparative Venn diagram showing the number of zoobenthic families detected by each method. Intersections represent common species shared among methods. Abbreviations: CN-B (Chinese Nearshore Benthos Classification System Books), eDNA-B (eDNA metabarcoding in this experiment), and Morph-B (traditional morpho-taxonomic approaches).

Although eDNA metabarcoding has proven to be a powerful tool for biodiversity assessment, significant potential remains for optimizing this technology for monitoring benthic organisms. The identified zoobenthic families in the present study were compared not only with those identified by traditional morpho-taxonomic approaches in the same study area, but also with the classifications in the Chinese Nearshore Benthos Classification System Book [43], which provide the most comprehensive introduction to zoobenthos in China (Fig 5). On the one hand, eDNA metabarcoding revealed 118 unique zoobenthic families in the Yellow River Estuary Coastal Wetland, demonstrating that this technique offers a new perspective for understanding zoobenthic diversity. On the other hand, traditional morpho-taxonomic approaches identified 60 zoobenthic families, whereas eDNA metabarcoding detected only 11 of these families. Additionally, out of the 771 zoobenthic families documented in Chinese zoobenthos literature, eDNA metabarcoding identified 49 families in the Yellow River Estuary Coastal Wetland, while traditional morpho-taxonomic methods identified 22 families. These findings suggest that a significant amount of biodiversity information remains undetected by eDNA metabarcoding.

This phenomenon can be partially attributed to the inefficiency of biological information collection. The specific survival patterns of different aquatic organisms influence their ecological niches and the spatial distribution of eDNA. Extraction of eDNA involves subsampling small volumes, often measured in grams. At this scale, the uneven distribution of eDNA, along with small organisms in the sediment, introduces considerable random sampling variability [27,44]. In contrast, morphological analysis typically involves sieving large volumes of sediment at specific mesh sizes. For example, Yang [19] collected benthic invertebrates from the Yellow River Estuary Coastal Wetland using a 100 cm × 35 cm sampling area at each point, which ensures that the analysis encompasses a significant portion of the community. In addition, the zoobenthic eDNA database is still incomplete, which limits the effective interpretation of biological information.

The effectiveness of eDNA depends heavily on the quality and completeness of reference databases. Without well-curated DNA barcodes, ambiguities in species identification may still arise, similar to challenges faced in morphology-based taxonomy. Thus, establishing a specialized eDNA database for zoobenthos is essential. In this study, a total of 981 families (975,690 reads) were identified at the taxonomic family level, comprising 174 benthic animal families (331,711 reads, 34.0%), 214 algal families (272,331 reads, 27.9%), 244 fungal families (36,975 reads, 3.8%), 16 parasitic families (1,032 reads, 0.1%), and 213 non-benthic protozoan families (333,615 reads, 34.2%). These data indicate a high proportion of non-benthic organisms, which increased the complexity of data analysis and potentially obscuring the true diversity of benthic animals. Additionally, the large number of "unclassified" sequences further highlights the inadequacy of current reference databases. To improve species identification accuracy, especially in specific ecosystems like the Yellow River Estuary, it is important to establish a localized eDNA database. This is particularly necessary as regional biodiversity may not be fully represented in global databases. This process should also be complemented by comprehensive benthic taxonomic inventories based on traditional methods, allowing for effective comparison and analysis of sequence data [45]. The raw sequencing data have already been uploaded to the NCBI database and can be accessed with the accession number PRJNA1134659.

### 4.2. Influence of habitat conditions on zoobenthic communities in the Yellow River Estuary

The Yellow River Estuary Coastal Wetland has undergone significant degradation due to agricultural reclamation, industrial pollution, and infrastructure development, which have adversely affected its ecological functions. However, decades of ecological restoration, including vegetation restoration, water resources management, pollution control, and micro-terrain shaping, contributed to the recovery of the wetland ecosystem, fostering rich species diversity [46].

Coastal wetlands feature waters with varying salinity levels, including freshwater, brackish water, and seawater. Salinity plays a crucial role in shaping these ecosystems. Brackish water with median salinity conditions exhibits the lowest species richness (Fig 3). This is due to fluctuating salinity levels, which create challenging conditions for many species [47]. As transitional zones, brackish waters limit the species that can adapt to both freshwater and marine conditions [48]. In addition to salinity, factors such as nutrient availability, sediment composition, and hydrodynamic conditions, along with human activities like habitat alteration and associated environmental pressures, significantly affect species diversity and community structure [49,50], often favoring more resilient species over those that are more sensitive [51]. Nevertheless, many marine species use brackish waters as nurseries for their juvenile stages [52]. These areas serve as crucial migratory pathways, contributing to dynamic but intermediate levels of species richness. Zoobenthic communities are highly sensitive to environmental stressors, such as pollution and habitat alterations, which can cause significant shifts in species composition. Studies from the Ganga River and the Damodar and Subarnarekha Rivers have shown that macrozoobenthic communities respond to various stressors, including pollution and habitat changes, by shifting towards species that are more tolerant of these conditions [53,54]. For instance, pollution-tolerant species like *Limnodrilus* tend to increase in abundance in areas impacted by industrial discharge, where they can outcompete more sensitive species. In contrast, species that are more sensitive to environmental changes, such as those with specific habitat requirements, may decline in these impacted areas [55]. This pattern of community restructuring highlights the resilience of certain zoobenthic taxa in the face of anthropogenic stressors. Additionally, indicator species are essential for evaluating wetland restoration. For instance, Chaetonotidae thrive in nutrient- and oxygen-rich environments, correlating with TOC and DO levels, while Monhysteridae, Dolichomacrostomidae, and Desmodoridae adapt to low-organic, low-oxygen conditions [56–60]. Their presence or absence reflects the region’s ecological health and highlights zoobenthic community dynamics in response to environmental changes. Furthermore, salinity has a significant impact on zoobenthic diversity. Species such as Naididae and Polycystididae exhibit positive correlations with salinity, inhabiting diverse environments from freshwater to marine conditions [61–64]. These species demonstrate strong adaptability to fluctuating salinity levels, which is crucial in an environment like the Yellow River Delta, where salinity varies across different zones. Conversely, families like Xyalidae and Chromadoridae are more sensitive to pH, preferring slightly alkaline environments [65,66]. These dynamics highlight the complex responses of zoobenthic communities to environmental fluctuations, suggesting their potential as bioindicators of salinity and other related stressors.

At a more specific level, the effect of salinity on zoobenthic genera is influenced by the composition of specific ions (e.g., Mg^2^⁺, Ca^2^⁺) (Fig 4). Elevated levels of these ions are essential for the health and diversity of certain taxa, such as *Longipedia* and *Halicyclops*, which show positive correlations with Mg^2^⁺ and Ca^2^⁺ [67]. These ions support crucial physiological functions, such as bone and shell formation, enzyme activity, and osmotic regulation. Other genera, like *Itaipusa*, benefit from the protection and stability provided by ion-rich sediments [68]. In contrast, changes in anion concentrations have a less pronounced impact on community structures [67]. Genera such as *Stronglostoma* and *Limnodrilus* show positive correlations with TOC and NH₄⁺, indicating their preference for nutrient-rich environments that support decomposition and nutrient cycling [69–71]. Moerisia, on the other hand, exhibits a negative correlation with TOC due to its sensitivity to organic pollution [72].

## 5. Conclusions

This study employed eDNA metabarcoding to reveal the biodiversity and distribution of zoobenthos in the ecological water replenishment area of Yellow River Estuary Coastal Wetland. This method identified 174 zoobenthic families, offering a broader and more comprehensive understanding of the ecosystem compared to traditional morpho-taxonomic approaches. Environmental factors such as salinity, cations, and organic matter significantly influence the composition and distribution of zoobenthos. These findings emphasize the importance of using advanced molecular techniques to assess biodiversity and ecological health in coastal wetlands. They provide new insights into the ecological dynamics and environmental stressors affecting zoobenthic communities under ecological management.

## Supporting information

S1 Fig Taxonomy and list of the 10 most abundant zoobenthic families. The columns in the figure represent the taxonomic levels from left to right: Domain, phylum, class, order, and family.
(TIF)

S2 Fig Network roles of analyzing module feature at genus level. Scatter plot of within-module connectivity (Zi) and among-module connectivity (Pi) showing the distribution of species based on their topological roles. Nodes are categorized into module hubs (Zi > 2.5 and Pi < 0.62), connectors (Zi < 2.5 and Pi > 0.62), network hubs (Zi > 2.5 and Pi > 0.62), and peripheral nodes (Zi < 2.5 and Pi < 0.62).
(TIF)

S1 Table Longitude and latitude coordinates of sampling sites. (DOCX)

S2 Table Taxonomic list of identified zoobenthos. (DOCX)

S1 Graphical abstract (TIF)
