# Supplementary figures and images for: Biodiversity and distribution of zoobenthos in the ecological water replenishment area of the Yellow River estuary coastal wetland revealed by eDNA metabarcoding
Source: PLoS One. 2024 Dec 18;19(12):e0315346. doi: 10.1371/journal.pone.0315346 (PMC11654974; doi:10.1371/journal.pone.0315346)

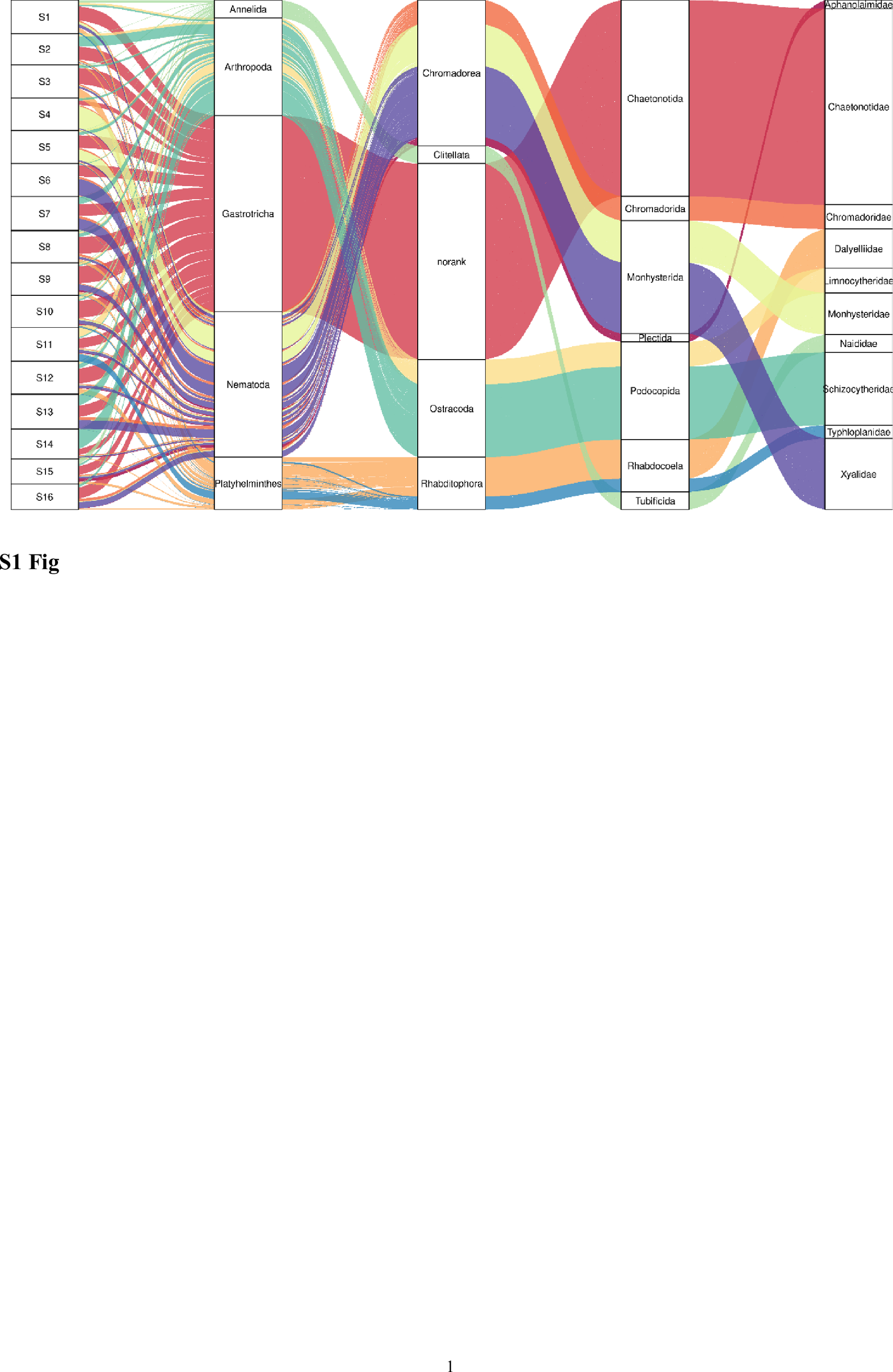

Supplement: S1 Fig — The columns in the figure represent the taxonomic levels from left to right: Domain, phylum, class, order, and family. (TIF) [file pone.0315346.s001.tif]

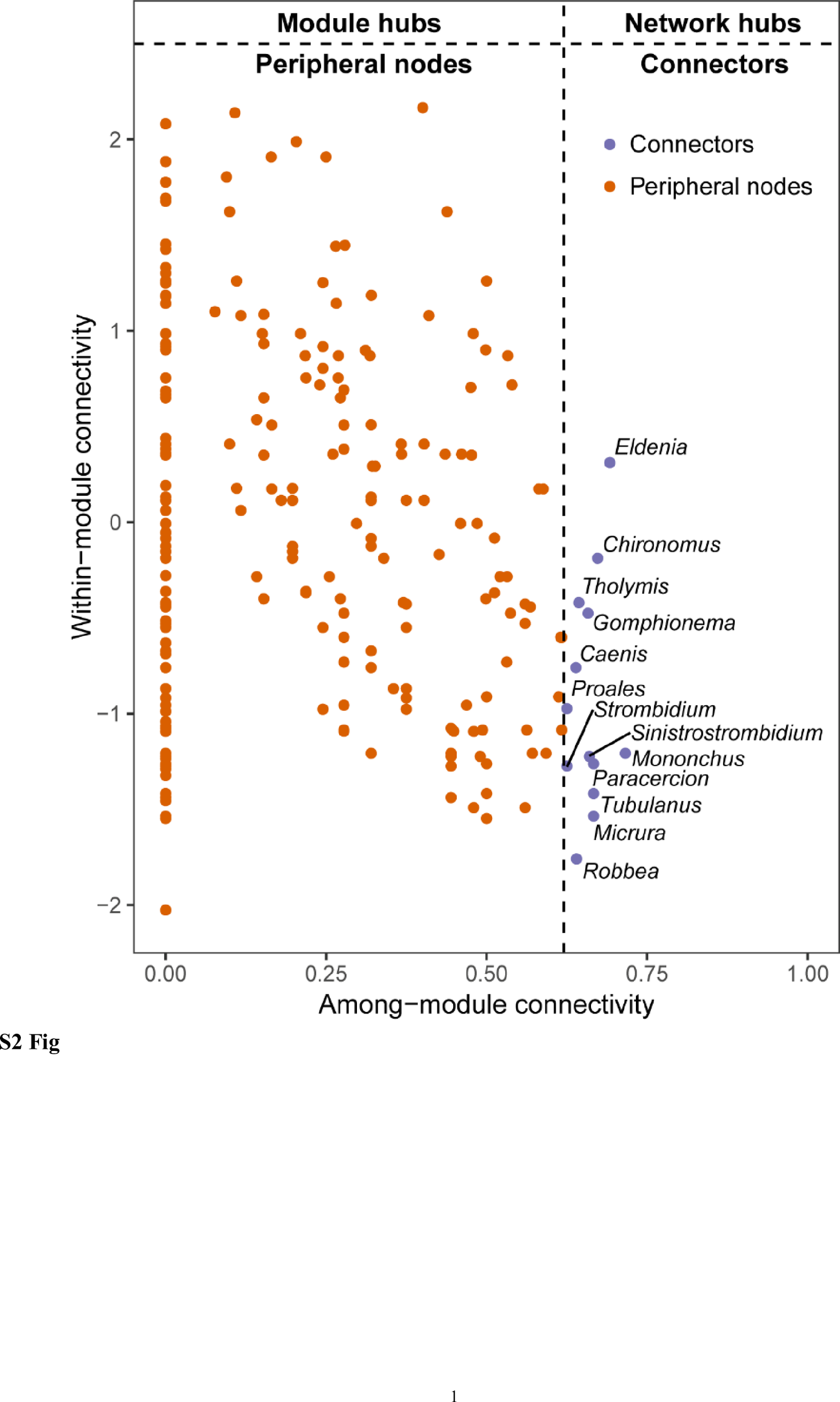

Supplement: S2 Fig — Scatter plot of within-module connectivity (Zi) and among-module connectivity (Pi) showing the distribution of species based on their topological roles. Nodes are categorized into module hubs (Zi > 2.5 and Pi < 0.62), connectors (Zi < 2.5 and Pi > 0.62), network hubs (Zi > 2.5 and Pi > 0.62), and peripheral nodes (Zi < 2.5 and Pi < 0.62). (TIF) [file pone.0315346.s002.tif]

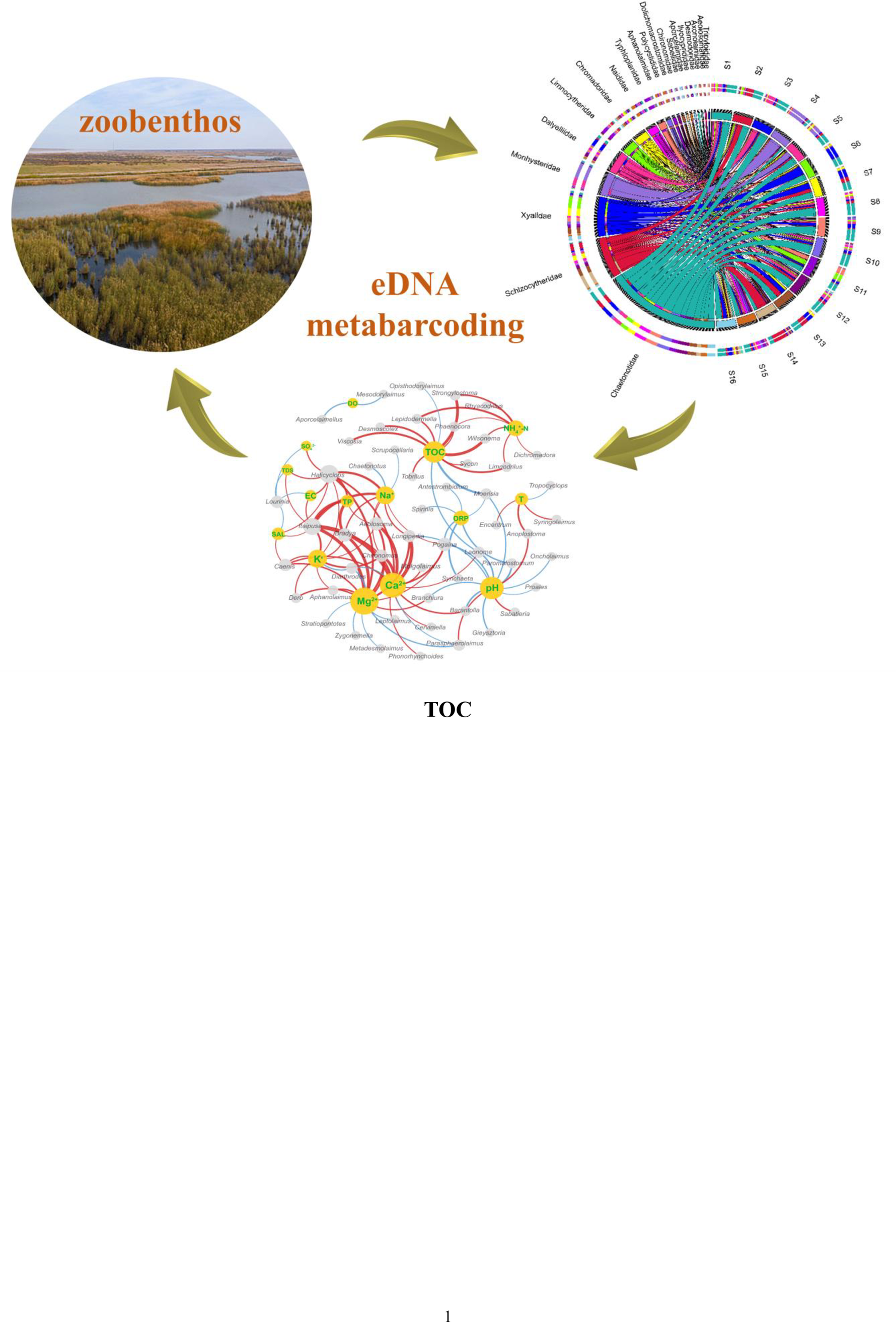

Supplement: S1 Graphical abstract — (TIF) [file pone.0315346.s005.tif]
