# Supplementary material for: Biodiversity and distribution of zoobenthos in the ecological water replenishment area of the Yellow River estuary coastal wetland revealed by eDNA metabarcoding
Source: PLoS One. 2024 Dec 18;19(12):e0315346. doi: 10.1371/journal.pone.0315346 (PMC11654974; doi:10.1371/journal.pone.0315346)
Supplement: S1 Table — (DOCX) [file pone.0315346.s003.docx]

**S1 Table**

| **Sampling site** | **Longitude (E)** | **Latitude (N)** |
| --- | --- | --- |
| S1 | 118.75662 | 38.06572 |
| S2 | 118.74392 | 38.02027 |
| S3 | 119.00132 | 37.80922 |
| S4 | 119.04454 | 37.83288 |
| S5 | 119.05696 | 37.81200 |
| S6 | 119.14227 | 37.77123 |
| S7 | 119.17046 | 37.75358 |
| S8 | 119.19571 | 37.77526 |
| S9 | 119.20715 | 37.78547 |
| S10 | 119.01665 | 37.74873 |
| S11 | 119.02655 | 37.75885 |
| S12 | 119.04939 | 37.74578 |
| S13 | 119.06117 | 37.75575 |
| S14 | 119.11778 | 37.72078 |
| S15 | 119.14065 | 37.71499 |
| S16 | 119.18904 | 37.71716 |
