# Supplementary material for: Biodiversity and distribution of zoobenthos in the ecological water replenishment area of the Yellow River estuary coastal wetland revealed by eDNA metabarcoding
Source: PLoS One. 2024 Dec 18;19(12):e0315346. doi: 10.1371/journal.pone.0315346 (PMC11654974; doi:10.1371/journal.pone.0315346)
Supplement: S2 Table — (DOCX) [file pone.0315346.s004.docx]

**S2 Table**

| **Domain** | **Phylum** | **Class** | **Order** | **Family** |
| --- | --- | --- | --- | --- |
| Eukaryota | Annelida | Clitellata | Crassiclitellata | Aeolosomatidae |
| Eukaryota | Annelida | Clitellata | Hirudinida | Piscicolidae |
| Eukaryota | Annelida | Clitellata | Tubificida | Naididae |
| Eukaryota | Annelida | Clitellata | Tubificida | Phreodrilidae |
| Eukaryota | Annelida | Polychaeta | Echiuroidea | Thalassematidae |
| Eukaryota | Annelida | Polychaeta | Eunicida | Amphinomidae |
| Eukaryota | Annelida | Polychaeta | Phyllodocida | Hesionidae |
| Eukaryota | Annelida | Polychaeta | Phyllodocida | Hisionidae |
| Eukaryota | Annelida | Polychaeta | Sabellida | Sabellidae |
| Eukaryota | Annelida | Polychaeta | Spionida | Spionidae |
| Eukaryota | Annelida | Polychaeta | Terebellida | Cirratulidae |
| Eukaryota | Annelida | Polychaeta | norank | Capitellidae |
| Eukaryota | Annelida | Polychaeta | norank | Chaetopteridae |
| Eukaryota | Annelida | Polychaeta | norank | Opheliidae |
| Eukaryota | Arthropoda | Hexanauplia | Cyclopoida | Archinotodelphyidae |
| Eukaryota | Arthropoda | Hexanauplia | Cyclopoida | Cyclopettidae |
| Eukaryota | Arthropoda | Hexanauplia | Cyclopoida | Cyclopidae |
| Eukaryota | Arthropoda | Hexanauplia | Cyclopoida | Cyclopinidae |
| Eukaryota | Arthropoda | Hexanauplia | Cyclopoida | Schminkepinellidae |
| Eukaryota | Arthropoda | Hexanauplia | Harpacticoida | Aegisthidae |
| Eukaryota | Arthropoda | Hexanauplia | Harpacticoida | Ameiridae |
| Eukaryota | Arthropoda | Hexanauplia | Harpacticoida | Canthocamptidae |
| Eukaryota | Arthropoda | Hexanauplia | Harpacticoida | Dactylopusiidae |
| Eukaryota | Arthropoda | Hexanauplia | Harpacticoida | Darcythompsoniidae |
| Eukaryota | Arthropoda | Hexanauplia | Harpacticoida | Ectinosomatidae |
| Eukaryota | Arthropoda | Hexanauplia | Harpacticoida | Harpacticidae |
| Eukaryota | Arthropoda | Hexanauplia | Harpacticoida | Laophontidae |
| Eukaryota | Arthropoda | Hexanauplia | Harpacticoida | Louriniidae |
| Eukaryota | Arthropoda | Hexanauplia | Harpacticoida | Nannopodidae |
| Eukaryota | Arthropoda | Hexanauplia | Harpacticoida | Tisbidae |
| Eukaryota | Arthropoda | Hexanauplia | Polyarthra | Longipediidae |
| Eukaryota | Arthropoda | Insecta | Diptera | Chironomidae |
| Eukaryota | Arthropoda | Insecta | Diptera | Tipulidae |
| Eukaryota | Arthropoda | Insecta | Ephemeroptera | Caenidae |
| Eukaryota | Arthropoda | Insecta | Odonata | Coenagrionidae |
| Eukaryota | Arthropoda | Insecta | Odonata | Libellulidae |
| Eukaryota | Arthropoda | Malacostraca | Amphipoda | Hyalellidae |
| Eukaryota | Arthropoda | Malacostraca | Bathynellacea | Parabathynellidae |
| Eukaryota | Arthropoda | Malacostraca | Decapoda | Eriphiidae |
| Eukaryota | Arthropoda | Ostracoda | Podocopida | Candonidae |
| Eukaryota | Arthropoda | Ostracoda | Podocopida | Cyclocyprididae |
| Eukaryota | Arthropoda | Ostracoda | Podocopida | Cyprididae |
| Eukaryota | Arthropoda | Ostracoda | Podocopida | Ilyocyprididae |
| Eukaryota | Arthropoda | Ostracoda | Podocopida | Limnocytheridae |
| Eukaryota | Arthropoda | Ostracoda | Podocopida | Loxoconchidae |
| Eukaryota | Arthropoda | Ostracoda | Podocopida | Schizocytheridae |
| Eukaryota | Bryozoa | Gymnolaemata | Cheilostomatida | Candidae |
| Eukaryota | Bryozoa | Gymnolaemata | Cheilostomatida | Celleporidae |
| Eukaryota | Bryozoa | Gymnolaemata | Ctenostomatida | Walkeriidae |
| Eukaryota | Bryozoa | Phylactolaemata | norank | Plumatellidae |
| Eukaryota | Cercozoa | Thecofilosea | norank | Ebriacea |
| Eukaryota | Ciliophora | Oligohymenophorea | norank | Schizocaryidae |
| Eukaryota | Ciliophora | Spirotrichea | norank | Deviatidae |
| Eukaryota | Ciliophora | Spirotrichea | norank | Strombidiidae |
| Eukaryota | Cnidaria | Anthozoa | Actiniaria | Actiniidae |
| Eukaryota | Cnidaria | Anthozoa | Actiniaria | Actinostolidae |
| Eukaryota | Cnidaria | Anthozoa | Actiniaria | Aiptasiidae |
| Eukaryota | Cnidaria | Anthozoa | Actiniaria | Diadumenidae |
| Eukaryota | Cnidaria | Anthozoa | Actiniaria | Edwardsiidae |
| Eukaryota | Cnidaria | Anthozoa | Scleralcyonacea | Pennatulidae |
| Eukaryota | Cnidaria | Anthozoa | Scleralcyonacea | Protoptilidae |
| Eukaryota | Cnidaria | Anthozoa | Scleralcyonacea | Virgulariidae |
| Eukaryota | Cnidaria | Anthozoa | Zoantharia | Epizoanthidae |
| Eukaryota | Cnidaria | Anthozoa | Zoantharia | Hydrozoanthidae |
| Eukaryota | Cnidaria | Anthozoa | Zoantharia | Parazoanthidae |
| Eukaryota | Cnidaria | Hydrozoa | Anthoathecata | Bougainvilliidae |
| Eukaryota | Cnidaria | Hydrozoa | Anthoathecata | Cordylophoridae |
| Eukaryota | Cnidaria | Hydrozoa | Anthoathecata | Corynidae |
| Eukaryota | Cnidaria | Hydrozoa | Anthoathecata | Hydractiniidae |
| Eukaryota | Cnidaria | Hydrozoa | Anthoathecata | Hydridae |
| Eukaryota | Cnidaria | Hydrozoa | Anthoathecata | Moerisiidae |
| Eukaryota | Cnidaria | Hydrozoa | Anthoathecata | Oceaniidae |
| Eukaryota | Cnidaria | Hydrozoa | Anthoathecata | Pandeidae |
| Eukaryota | Cnidaria | Hydrozoa | Anthoathecata | Sphaerocorynidae |
| Eukaryota | Cnidaria | Hydrozoa | Anthoathecata | Stylasteridae |
| Eukaryota | Cnidaria | Hydrozoa | Leptothecata | Hebellidae |
| Eukaryota | Cnidaria | Hydrozoa | Siphonophorae | Diphyidae |
| Eukaryota | Cnidaria | Hydrozoa | Siphonophorae | Physophoridae |
| Eukaryota | Cnidaria | Hydrozoa | Siphonophorae | Pyrostephidae |
| Eukaryota | Cnidaria | Hydrozoa | Trachymedusae | Rhopalonematidae |
| Eukaryota | Cnidaria | Scyphozoa | Semaeostomeae | Cyaneidae |
| Eukaryota | Entoprocta | norank | norank | Barentsiidae |
| Eukaryota | Foraminifera | norank | Rotaliida | Rotaliidae |
| Eukaryota | Gastrotricha | norank | Chaetonotida | Chaetonotidae |
| Eukaryota | Gastrotricha | norank | Chaetonotida | Dasydytidae |
| Eukaryota | Gastrotricha | norank | Chaetonotida | Neogosseidae |
| Eukaryota | Mollusca | Bivalvia | Cardiida | Semelidae |
| Eukaryota | Mollusca | Bivalvia | Cardiida | Tellinidae |
| Eukaryota | Mollusca | Bivalvia | Mytilida | Mytilidae |
| Eukaryota | Mollusca | Bivalvia | Ostreida | Ostreidae |
| Eukaryota | Mollusca | Gastropoda | Littorinimorpha | Assimineidae |
| Eukaryota | Mollusca | Gastropoda | Littorinimorpha | Caecidae |
| Eukaryota | Mollusca | Gastropoda | norank | Lymnaeidae |
| Eukaryota | Nematoda | Chromadorea | Araeolaimida | Axonolaimidae |
| Eukaryota | Nematoda | Chromadorea | Araeolaimida | Comesomatidae |
| Eukaryota | Nematoda | Chromadorea | Araeolaimida | Diplopeltidae |
| Eukaryota | Nematoda | Chromadorea | Chromadorida | Chromadoridae |
| Eukaryota | Nematoda | Chromadorea | Chromadorida | Cyatholaimidae |
| Eukaryota | Nematoda | Chromadorea | Desmodorida | Desmodoridae |
| Eukaryota | Nematoda | Chromadorea | Desmoscolecida | Desmoscolecidae |
| Eukaryota | Nematoda | Chromadorea | Microlaimida | Microlaimidae |
| Eukaryota | Nematoda | Chromadorea | Microlaimida | Molgolaimidae |
| Eukaryota | Nematoda | Chromadorea | Monhysterida | Linhomoeidae |
| Eukaryota | Nematoda | Chromadorea | Monhysterida | Monhysteridae |
| Eukaryota | Nematoda | Chromadorea | Monhysterida | Sphaerolaimidae |
| Eukaryota | Nematoda | Chromadorea | Monhysterida | Xyalidae |
| Eukaryota | Nematoda | Chromadorea | Plectida | Aphanolaimidae |
| Eukaryota | Nematoda | Chromadorea | Plectida | Haliplectidae |
| Eukaryota | Nematoda | Chromadorea | Plectida | Leptolaimidae |
| Eukaryota | Nematoda | Chromadorea | Plectida | Plectidae |
| Eukaryota | Nematoda | Chromadorea | Rhabditida | Strongyloididae |
| Eukaryota | Nematoda | Enoplea | Dorylaimida | Aetholaimidae |
| Eukaryota | Nematoda | Enoplea | Dorylaimida | Aporcelaimidae |
| Eukaryota | Nematoda | Enoplea | Dorylaimida | Dorylaimidae |
| Eukaryota | Nematoda | Enoplea | Dorylaimida | Pararhyssocolpidae |
| Eukaryota | Nematoda | Enoplea | Dorylaimida | Qudsianematidae |
| Eukaryota | Nematoda | Enoplea | Enoplida | Anoplostomatidae |
| Eukaryota | Nematoda | Enoplea | Enoplida | Ironidae |
| Eukaryota | Nematoda | Enoplea | Enoplida | Oncholaimidae |
| Eukaryota | Nematoda | Enoplea | Enoplida | Oxystominidae |
| Eukaryota | Nematoda | Enoplea | Enoplida | Rhabdolaimidae |
| Eukaryota | Nematoda | Enoplea | Enoplida | Tripyloididae |
| Eukaryota | Nematoda | Enoplea | Mermithida | Mermithidae |
| Eukaryota | Nematoda | Enoplea | Mononchida | Mononchidae |
| Eukaryota | Nematoda | Enoplea | Mononchida | Mylonchulidae |
| Eukaryota | Nematoda | Enoplea | Triplonchida | Tobrilidae |
| Eukaryota | Nematoda | Enoplea | Triplonchida | Tripylidae |
| Eukaryota | Nemertea | Enopla | Monostilifera | Tetrastemmatidae |
| Eukaryota | Nemertea | Palaeonemertea | Tubulaniformes | Tubulanidae |
| Eukaryota | Nemertea | Pilidiophora | Heteronemertea | Lineidae |
| Eukaryota | Perkinsozoa | norank | norank | Parviluciferaceae |
| Eukaryota | Placozoa | Uniplacotomia | Trichoplacea | Trichoplacidae |
| Eukaryota | Platyhelminthes | Catenulida | norank | Stenostomidae |
| Eukaryota | Platyhelminthes | Cestoda | Caryophyllidea | Lytocestidae |
| Eukaryota | Platyhelminthes | Monogenea | Dactylogyridea | Ancylodiscoididae |
| Eukaryota | Platyhelminthes | Monogenea | Dactylogyridea | Ancyrocephalidae |
| Eukaryota | Platyhelminthes | Monogenea | Dactylogyridea | Dactylogyridae |
| Eukaryota | Platyhelminthes | Monogenea | Dactylogyridea | Pseudodactylogyridae |
| Eukaryota | Platyhelminthes | Rhabditophora | Macrostomida | Dolichomacrostomidae |
| Eukaryota | Platyhelminthes | Rhabditophora | Macrostomida | Macrostomidae |
| Eukaryota | Platyhelminthes | Rhabditophora | Rhabdocoela | Dalyelliidae |
| Eukaryota | Platyhelminthes | Rhabditophora | Rhabdocoela | Koinocystididae |
| Eukaryota | Platyhelminthes | Rhabditophora | Rhabdocoela | Placorhynchidae |
| Eukaryota | Platyhelminthes | Rhabditophora | Rhabdocoela | Polycystididae |
| Eukaryota | Platyhelminthes | Rhabditophora | Rhabdocoela | Provorticidae |
| Eukaryota | Platyhelminthes | Rhabditophora | Rhabdocoela | Trigonostomidae |
| Eukaryota | Platyhelminthes | Rhabditophora | Rhabdocoela | Typhloplanidae |
| Eukaryota | Platyhelminthes | Trematoda | Plagiorchiida | Collyriclidae |
| Eukaryota | Porifera | Calcarea | Clathrinida | Clathrinidae |
| Eukaryota | Porifera | Calcarea | Clathrinida | Leucettidae |
| Eukaryota | Porifera | Calcarea | Leucosolenida | Grantiidae |
| Eukaryota | Porifera | Calcarea | Leucosolenida | Leucosoleniidae |
| Eukaryota | Porifera | Calcarea | Leucosolenida | Sycettidae |
| Eukaryota | Porifera | Demospongiae | Agelasida | Agelasidae |
| Eukaryota | Porifera | Demospongiae | Biemnida | Biemnidae |
| Eukaryota | Porifera | Demospongiae | Bubarida | Dictyonellidae |
| Eukaryota | Porifera | Demospongiae | Haplosclerida | Chalinidae |
| Eukaryota | Porifera | Demospongiae | Poecilosclerida | Coelosphaeridae |
| Eukaryota | Porifera | Demospongiae | Sphaerocladina | Vetulinidae |
| Eukaryota | Porifera | Demospongiae | Spongillida | Spongillidae |
| Eukaryota | Porifera | Demospongiae | Suberitida | Halichondriidae |
| Eukaryota | Porifera | Demospongiae | Suberitida | Suberitidae |
| Eukaryota | Porifera | Demospongiae | Verongiida | Aplysinidae |
| Eukaryota | Preaxostyla | norank | norank | Paratrimastigidae |
| Eukaryota | Rotifera | Eurotatoria | Flosculariaceae | Flosculariidae |
| Eukaryota | Rotifera | Eurotatoria | Ploima | Brachionidae |
| Eukaryota | Rotifera | Eurotatoria | Ploima | Dicranophoridae |
| Eukaryota | Rotifera | Eurotatoria | Ploima | Lecanidae |
| Eukaryota | Rotifera | Eurotatoria | Ploima | Lepadellidae |
| Eukaryota | Rotifera | Eurotatoria | Ploima | Notommatidae |
| Eukaryota | Rotifera | Eurotatoria | Ploima | Proalidae |
| Eukaryota | Rotifera | Eurotatoria | Ploima | Synchaetidae |
| Eukaryota | Tardigrada | Eutardigrada | Parachela | Doryphoribiidae |
| Eukaryota | Xenacoelomorpha | norank | Acoela | Otocelididae |
